# Supplementary material for: Mental imagery and breathing exercises integrated into a standardized warm-up routine enhance sympathetic activation and optimize muscular performance in firefighters
Source: PLoS One. 2025 Dec 3;20(12):e0337431. doi: 10.1371/journal.pone.0337431 (PMC12674522; doi:10.1371/journal.pone.0337431)
Supplement: Attachment — Submitted filename: Response to reviewers.docx [file pone.0337431.s003.docx]

**Response to reviewers: for the sake of clarity, all responses to the reviewer are provided in bold font, immediately following each of their remarks, comments, or questions listed below.**

1. Is the manuscript technically sound, and do the data support the conclusions?

The manuscript must describe a technically sound piece of scientific research with data that supports the conclusions. Experiments must have been conducted rigorously, with appropriate controls, replication, and sample sizes. The conclusions must be drawn appropriately based on the data presented.

Reviewer #1: Yes

2. Has the statistical analysis been performed appropriately and rigorously?

Reviewer #1: Yes

3. Have the authors made all data underlying the findings in their manuscript fully available?

The [PLOS Data policy](http://track.editorialmanager.com/CL0/http:%2F%2Fwww.plosone.org%2Fstatic%2Fpolicies.action%23sharing/1/010f0197f03fedc3-a56b7ae4-3464-4fdb-8ad9-292d3910a21a-000000/hWFVDQb_lewe4hZ00LOi7rikosNEqlVniHzdo5oHBUs=219) requires authors to make all data underlying the findings described in their manuscript fully available without restriction, with rare exception (please refer to the Data Availability Statement in the manuscript PDF file). The data should be provided as part of the manuscript or its supporting information, or deposited to a public repository. For example, in addition to summary statistics, the data points behind means, medians and variance measures should be available. If there are restrictions on publicly sharing data—e.g. participant privacy or use of data from a third party—those must be specified.

Reviewer #1: Yes

4. Is the manuscript presented in an intelligible fashion and written in standard English?

PLOS ONE does not copyedit accepted manuscripts, so the language in submitted articles must be clear, correct, and unambiguous. Any typographical or grammatical errors should be corrected at revision, so please note any specific errors here.

Reviewer #1: Yes

5. Review Comments to the Author

Please use the space provided to explain your answers to the questions above. You may also include additional comments for the author, including concerns about dual publication, research ethics, or publication ethics. (Please upload your review as an attachment if it exceeds 20,000 characters)

Reviewer #1: The present study investigated the effects of mental imagery and breathing added to a conventional warm-up on the sympathetic activation and muscle force and endurance. Some major concerns should be addressed to clarify the rationale (introduction), to justify the methods (2 repeated warm-up sessions for example), to clarify the statistics (e.g. huge variability) and to make the discussion clear. Please see below.

**Title and Abstract.**

The title is clear and the abstract almost adequately describe the study. The methods (e.g., the 2 experimental groups? the muscle group?) and results (e.g., physical performances?) should be developed for clarity. The last sentence is very general and not really helpful. Please consider rephrasing and giving a clear conclusion related to the present findings.

**We have modified the abstract accordingly as follows (highlighted in yellow) to address these comments:**

***“******The capacity of firefighters to consistently mobilize their full physical potential during duty shifts is of paramount importance. One promising approach to achieving this goal involves developing operational protocols that effectively activate the sympathetic nervous system and vagal regulation prior to each professional action. This study investigated the effects of combining mental imagery and conscious breathing exercises with a conventional and standard warm-up on cardiac modulations, muscular strength and endurance performance in 34 firefighters randomly assigned to one of two groups: a control group (n=17), which performed only the conventional and standard warm-up, and an experimental group (n=17), which performed the standard warm-up in combination with mental imagery and breathing exercises. The results showed that incorporating such psychophysiological techniques into a conventional warm-up during repeated physical efforts optimizes sympathetic spectral power, heart rate responses, and physical performances, including maximal voluntary isometric handgrip contraction, maximum push-ups, and the maximal abdominal plank duration. Altogether, these findings indicate that brief mental imagery and controlled breathing exercises incorporated into standard warm-up routines effectively preserve muscular performance under repeated exertion while enhancing sympathetic activation, offering a practical and immediately applicable strategy to optimize firefighters’ operational readiness.”***

**Introduction.**

L51. high level of cardiac stress is too restrictive.

**We have modified accordingly as follows (highlighted in yellow) to address these comments:**

***“Firefighters are required to operate in hostile*** ***environments that involve multiple highly demanding physical tasks combined with highly engaging emotional contexts, both of which elicit substantial cardiovascular strain—characterized by pronounced elevations in heart rate—while rescuing victims and combating fires, often at the risk of their own lives (Gendron et al., 2019).”***

L53. What does 'job performance' mean?

**We have revised this sentence to specify the operational components of job performance in the firefighting context, namely the ability to effectively and safely perform physically demanding firefighting tasks (e.g., victim rescue, hose handling, ladder climbing, equipment carrying), which are commonly used as validated occupational performance indicators in the scientific literature.**

**We have modified accordingly as follows (highlighted in yellow) to address these comments:**

***“A high level of physical fitness, muscular endurance, and muscular strength has been*** ***associated*** ***with improved performance in operational firefighting tasks—such as victim rescue, hose handling, ladder climbing, and equipment carrying—as well as with a reduced risk of injury (Johnson et al., 2022; Michaelides et al., 2008; Mattila et al., 2007).”***

L67-L75. The message behind this paragraph is unclear. What is the link with the preceding and following paragraphs.

**We agree that the original paragraph -L67-75- on the neurophysiological definitions of muscular strength and endurance was too artificial and insufficiently connected to the surrounding sections. To address this, we have revised the text to explicitly link these definitions to the mechanisms through which fatigue impacts firefighters’ physical performance capacity, and to the rationale for exploring countermeasures that target both peripheral and central determinants of performance. This clarification aims now to create a smoother logical transition from the description of operational fatigue factors to the presentation of our psychophysiological intervention strategy.**

**We have modified accordingly as follows (highlighted in yellow) to address these comments:**

***“******From a neurophysiological perspective, muscular strength refers to the capacity to generate force during a single, brief, maximal voluntary contraction of a muscle at rest (Lewis & Haller, 1991), whereas dynamic and isometric muscular endurance can be defined as the ability to sustain or repeat muscular effort across multiple dynamic movements or during prolonged postural maintenance (Larsson & Karlsson, 1978). Importantly, these physical capacities are governed not only by peripheral musculoskeletal factors but are also strongly modulated by neural mechanisms, including the central nervous system (CNS) and the autonomic nervous system (ANS), which regulates heart rate (HR) and heart rate variability (HRV). This neurophysiological regulation plays a crucial role in determining how fatigue develops and how performance is maintained under operational constraints (Schmitt et al., 2013).”***

L81-82. It is unclear why activating the sympathetic system may be beneficial. The optimization of warm-up routines is easier to understand. It can be achieved while lowering fatigue associated with the different warm-up exercises and while optimizing some physiological systems (for example the sympathetic). But the usefulness of the sympathetic system should be made clearer.

**Activating the sympathetic nervous system before exertion primes cardiovascular and neuromuscular responses, thereby may optimize warm-up efficiency and subsequent performance (Tran et al., 2001; Csala et al., 2021).**

**We have added accordingly the following sentences (highlighted in yellow) to address these comments:**

***“From a neurophysiological perspective, muscular strength refers to the capacity to generate force during a single, brief, maximal voluntary contraction of a muscle at rest (Lewis & Haller, 1991), whereas dynamic and isometric muscular endurance can be defined as the ability to sustain or repeat muscular effort across multiple dynamic movements or during prolonged postural maintenance (Larsson & Karlsson, 1978). Importantly, these physical capacities are governed not only by peripheral musculoskeletal factors but are also strongly modulated by neural mechanisms, including the central nervous system (CNS) and the autonomic nervous system (ANS), which regulates heart rate (HR) and heart rate variability (HRV). This neurophysiological regulation plays a crucial role in determining how fatigue develops and how performance is maintained under operational constraints (Schmitt et al., 2013******). In this context, activating the sympathetic nervous system prior to strenuous effort enhances cardiovascular readiness and facilitates greater muscle recruitment, thereby improving strength and endurance performance (Tran et al., 2001; Csala et al., 2021). Such pre-activation primes the body to meet acute energetic demands by increasing heart rate, blood flow, and neuromuscular excitability, thereby potentially optimizing warm-up efficacy.***

***Within this framework, and without modifying firefighters' work schedules (in France, in the present study), two potential countermeasures could help prevent […]”***

L89-95. Authors seem to indicate these effects are related to the autonomic nervous system. Please explain or clarify. Generally speaking, the effect of motor imagery on the autonomic nervous system is unclear. Similarly, the link between this autonomic system and strength is unclear.

*“effect of motor imagery on the autonomic nervous system”:* **Motor imagery can influence the autonomic nervous system by modulating heart rate and heart rate variability, reflecting shifts in sympathovagal balance. Such effects suggest that imagery not only primes corticospinal pathways but may also contribute to autonomic adjustments that facilitate physical readiness (Decety, 1996; Grosprêtre et al., 2016; Bouguetoch et al., 2021).**

*“link between this autonomic system and strength”:* **Sympathetic nervous system activation enhances muscular strength and endurance by increasing heart rate, blood pressure, and muscle blood flow, thereby facilitating oxygen delivery and metabolite clearance. In parallel, it augments neuromuscular excitability and motor unit recruitment, mechanisms that together support improved force production and resistance to fatigue (Lombardi et al., 1996; Tran et al., 2001; Csala et al., 2021; *Kazuo et al. 2000; Pinto et al. 2017*).**

**In line with this comment, we have now clarified the known effects of emotional visualization and motor imagery on the autonomic nervous system and on strength levels. We have added accordingly the following sentences (highlighted in yellow) to address these comments:**

***“******Firstly, certain mental training techniques, such as motor imagery (Bouguetoch et al., 2021) and emotional visualization (Coombes et al., 2009), have been identified as potential methods to enhance corticospinal excitability, thereby promoting muscle strength and endurance. Although the primary and well-established effects of motor imagery are neural and centrally mediated, several studies also suggested that it can influence autonomic responses, as evidenced by changes in heart rate and heart rate variability (Oishi et al. 2000; Peixeito Pinto et al. 2017 ).”***

**Added references:**

- **Peixoto Pinto T, Mello Russo Ramos M, Lemos T, Domingues Vargas C, Imbiriba LA. Is heart rate variability affected by distinct motor imagery strategies? Physiol Behav. 2017 Aug 1;177:189-195. doi: 10.1016/j.physbeh.2017.05.004**
- **Kazuo Oishi, Tatsuya Kasai, Takashi Maeshima, Autonomic Response Specificity during Motor Imagery, Journal of PHYSIOLOGICAL ANTHROPOLOGY and Applied Human Science, 2000, Volume 19, Issue 6, Pages 255-261**

L108-112. The hypothesis should also be clarified. Why less performance decline? The exercise considered should be defined here.

*“Why less performance decline”:* **When two maximal efforts are performed in succession (particularly those involving strength endurance to failure, such as the maximum number of push-ups or the maximal duration of the abdominal plank used in this study) the second performance is generally reduced due to both peripheral and mental fatigue induced by the first effort. This pattern was also observed in our previous work (Biéchy et al. 2021). Therefore, we hypothesized that, during the second bout of maximal testing, the experimental group (benefiting from the addition of psychophysiological practices to their usual warm-up) would experience a smaller decline in performance compared to the control group without such practices.**

**This situation of repeated high-intensity efforts is also of practical relevance, as it closely reflects the real working conditions of firefighters, who often perform successive interventions during their duty shifts with little or no opportunity for recovery.**

**We have modified accordingly the following sentences (highlighted in yellow) to address these comments:**

***“We hypothesized that firefighters performing a standard warm-up supplemented with mental imagery and breathing exercises would exhibit a smaller performance decline —or even performance enhancement— during repeated maximal efforts, including maximal voluntary isometric handgrip contraction, maximum number of push-ups, and maximal abdominal plank duration. These practices were expected to help counteract both central and peripheral fatigue while enhancing corticospinal excitability, cardiovascular readiness and sympathetic activation, compared with controls who performed only the conventional warm-up without psychophysiological techniques.”***

Methods.

L132-135. What is the rationale for considering 2 experimental groups and not considering a cross-over design? Please justify the sample size.

*“2 experimental groups and not considering a cross-over design:”* **Regarding the choice of design. We opted for two parallel groups rather than a cross-over because the intervention (mental imagery + controlled breathing integrated into the warm-up routine) is likely to produce carryover effects on both autonomic state (lingering sympathovagal modulation) and behavior (learning/expectancy and psyching-up skills), which a short washout would not reliably eliminate. In addition, our testing sequence already involves repeated maximal efforts (handgrip MVIC, maximal push-ups, maximal abdominal plank) that induce central and peripheral fatigue. In a cross-over this fatigue and recovery trajectory would confound period/sequence effects and require long washouts incompatible with firefighters’ duty schedules. And finally, the parallel-group design maximizes ecological validity, mirroring how such psychophysiological routines would be implemented operationally.**

*“Please justify the sample size”:* **A priori, we powered the Group × Session interaction of a mixed ANOVA on our primary outcomes (maximal performances and HR/HRV indices), targeting a medium effect size (f = 0.25–0.30), α = .05, power (1–β) = .80, correlation among repeated measures ≈ .50, non-sphericity = 1.0. This effect size was chosen because it corresponds to the magnitude generally observed in this type of psychophysiological intervention, and particularly in our previous studies (Biéchy et al., 2021; Jamous et al., 2024). Using G*Power (RM-ANOVA, between-factor), this yielded a required total sample of ≈ 26–32 participants. Anticipating ~10–15% attrition and occasional unusable HRV files, we planned n = 34 and achieved this target, with random allocation of 17 participants per group, of whom 28 were equipped with a heart rate monitor, which still meets our targeted statistical power.**

**We have added accordingly the following sentences (highlighted in yellow) to address these comments:**

***“******Based on G*Power calculations (f = 0.25–0.30), α = .05, and power = .80, a total of 28–32 participants was required, consistent with prior psychophysiological studies (Biéchy et al., 2021; Jamous et al., 2024). We therefore recruited 34 firefighters (age: 34.9 ± 6.9 years; height: 178 ± 4.2 cm; weight: 77.4 ± 5.8 kg; body mass index: 24.6 ± 1.1 kg/m2) volunteered for the experiment.”***

L146. How were volunteers during the 30-min rest (seated, lying...)?

**During the 30-min rest period, volunteers remained in a standing posture on both feet. All parameters potentially influencing the autonomic nervous system were strictly controlled and standardized across groups, including hydration status, restroom use, and the maintenance of this identical passive standing position.**

**We have modified accordingly to address these comments:**

***“Following this assessment, participants underwent a 30-minute rest period*** ***in a passive standing posture on both feet, during which all parameters potentially influencing autonomic function (hydration, restroom use, posture) were controlled and standardized. During this interval, 28 out of the 34 firefighters were randomly selected to be fitted with a heart rate monitor.”***

L147. Why only 28 with HRV measurements? What were not involved volunteers doing during these 15 minutes?

**Unfortunately, only 28 out of 34 participants could undergo HRV measurements because our equipment allowed a maximum of 28 sensors, and for operational reasons related to firefighters’ duty schedules, it was not possible to organize an additional complete experimental session. Nevertheless, since 34 volunteers had been successfully recruited, we decided to retain the six additional participants for the physical performance tests. During the HRV recording period, the non-equipped participants followed exactly the same procedures as the equipped ones.**

L149. Figure 1 indicates 15 minutes for HRV test (also L154).

**Yes, precisely. The standardized Schmitt Tilt Test (Schmitt et al., 2013) involves an 8-minute cardiac recording in the supine position followed by 7 minutes in the standing position, resulting in a total duration of 15 minutes.**

L154-155. HRV was evaluated after a single warm-up while muscle force/endurance were evaluated after 2 warm-up protocols. Have authors tested the potential bias? Were muscle properties similarly affected after a single or double warm-up procedure? Same question for HRV.

**There is no systematic bias in our protocol because both groups underwent identical warm-up procedures — namely, two warm-up sequences (before and after the HRV interval). It is conceivable, however, that the magnitude of physiological effects (e.g. on HRV or neuromuscular readiness) may differ slightly between the first vs. second warm-up, especially when underlying mechanisms are investigated in the discussion section; we will explicitly address this as a limitation in the revised manuscript (as recommended below by the reviewer).**

**We implemented the double warm-up because the 15-minute HRV assessment (supine + standing) tends to reduce baseline tone and readiness (i.e. induces mild parasympathetic rebound and drop in neuromuscular activation). To ensure that strength and endurance tests would start from a comparable physiological state across subjects, we re-warmed participants equally. This design choice also served a safety rationale: firefighters (professionals) could be at increased injury risk if asked to perform maximal efforts from a depressed neuromuscular state (Okobi et al 2022). Regarding decay of warm-up effects over time, the warm-up “heat and activation” benefits are transient, with reduction of neural facilitation and temperature benefits if a period of inactivity intervenes (i.e. necessitating re-warm-up) (Afonso et al. 2024).**

**And finaly, given operational constraints in their duty schedules, conducting a completely separate session was not feasible.**

**References:**

- **Okobi OE, Evbayekha EO, Ilechie E, Iroro J, Nwafor JN, Gandu Z, Shittu HO. A Meta-Analysis of Randomized Controlled Trials on the Effectiveness of Exercise Intervention in Preventing Sports Injuries. Cureus. 2022 Jun 20;14(6):e26123. doi: 10.7759/cureus.26123**
- **Afonso, J., Brito, J., Abade, E. *et al.* Revisiting the ‘Whys’ and ‘Hows’ of the Warm-Up: Are We Asking the Right Questions?. *Sports Med* 54, 23–30 (2024). https://doi.org/10.1007/s40279-023-01908-y**

L166, L170 and table 1. It is not table A1 but table 1. It is unclear if participants are doing mental imagery during or following the warm-up exercice. How did authors controlled the intensity of each exercices between CTRL and EXP. Did authors control the breathing and mental imagery? and how?

*“It is not table A1 but table 1”:* **We have modified accordingly.**

*“It is unclear if participants are doing mental imagery during or following the warm-up exercice”:* **Participants are doing mental imagery DURING the warm-up exercice. This was illustrated in Figure 1, or in the Table 1 (“*during the neck rotation exercises, participants were asked to voluntarily controlled their breathing”****)* **and this has now been clarified in the manuscript.**

**We have modified accordingly to address these comments:**

***“The EXP warm-up contents are composed by the same sequence of real muscle contractions as the CTRL group, to which are added*, *during the performance of the muscular exercises, the mental imagery practices and the breathing exercises* *that are specifically indicated in the right column.”***

“How did authors controlled the intensity of each exercices between CTRL and EXP”: **This was controlled by the Borg Rating of Perceived Exertion scale at the end of each exercise in both group and in combination with the inspection of the maximal heart rates achieved.**

“Did authors control the breathing and mental imagery? and how?”: **Respiratory rates were visually controlled for each participant across both groups. Motor imagery cannot be objectively controlled during the exercise without electrophysiological recordings; it could be only be assessed beforehand using specific questionnaires such as the MIQ-3 Short Form (Movement Imagery Questionnaire) (Robin et al., 2021) 🡪 This point has now been added in the new “Limitations” paragraph.**

L180. How many contractions? possible rest duration? Rest between this test and push-ups? Moreover, push-up is a form of dynamic plank. What is the influence of this pre-exercice. It can serve as a fatiguing exercice or as an additional warm-up exercice.

**If the trial was properly executed (no slipping of the handgrip and full compliance with the instructions, i.e., >99% of trials), the MVIC was measured in a single attempt. Therefore, no rest period was required. The rest interval between this test and the push-ups was limited to the setup time, approximately 3 minutes.**

“Moreover, push-up is a form of dynamic plank. What is the influence of this pre-exercice. It can serve as a fatiguing exercice or as an additional warm-up exercice:” **We agree that the push-up test may be considered as a pre-fatiguing exercise for the dynamic plank. Noteworthy is that as it is a maximal series performed to exhaustion, it cannot truly be considered as a warm-up. Indeed, the purpose of a warm-up is to balance the increase in body temperature with the limitation of unnecessary energy expenditure. Anyway, we are fully confident that this does not affect the integrity of our experimental protocol, since the same testing sequence was applied identically across sessions and across groups. Thus, all conditions were strictly standardized, and the only variable that differed between groups was the type of warm-up.**

L199. 6-min rest was observed between push-up and plank. This long rest could reduce the warm-up effects. Was the effect of this delay tested?

**We did not specifically test this rest-interval. Six minutes is a classical recovery period in the sport science literature for regaining readiness after a maximal strength muscular exercise. The residual effects of a warm-up are also known to last on average around 15 minutes, and therefore this 6-minute interval is well below that threshold (Afonso et al 2024). Moreover, our results are consistent with this assumption.**

**Reference:**

- **Afonso, J., Brito, J., Abade, E. *et al.* Revisiting the ‘Whys’ and ‘Hows’ of the Warm-Up: Are We Asking the Right Questions?. *Sports Med* 54, 23–30 (2024). https://doi.org/10.1007/s40279-023-01908-y**

L210. Conducting warm-up + mental imagery + controlled breathing could increase the (warm-up) perceived exertion. Why was RPE not evaluated following warm-up.

**First, the participants displayed physical capacities consistent with professional firefighting requirements, and thus superior to those of a sedentary population. As highly trained individuals exercising several times per week, the proposed warm-up represented only a basic effort, minimal and with no meaningful impact on perceived exertion. Similarly, the mental imagery load was considered negligible, as such practice is routinely integrated into their cognitive and motor drills. Controlled breathing exercises were also not regarded as an additional constraint, since firefighters are accustomed to regulating their breathing both during training and in operational contexts, such as when using self-contained breathing apparatus (SCBA) during fire interventions. Second, for operational reasons, the total duration of the experimental protocol could not be extended indefinitely. Third, while this remark is correct, it represents an additional question beyond the scientific questions and hypotheses initially formulated for this study. It is therefore a parallel issue, which may indeed be informative or explanatory, but does not constitute the primary scientific focus of the present work.**

**For these reasons, we did not consider it scientifically relevant to measure RPE scores at these specific time points.**

L223. How was respiratory rate measured?

**Respiratory rate was visually inspected by experimenters. This precision is now added in the manuscript.**

**Results.**

Please be consistant while writing pre/post or PRE/POST.

**We agree with this remark, and this point has now been addressed accordingly in the manuscript.**

Results and Figures. Figure should be modified to better show the * related to the 'performance' decrease in the control group. Also, was performance similar between groups post-warm-up? In the text, values during pre-tests are useless and redundant with figure 2.

“Results and Figures. Figure should be modified to better show the * related to the 'performance' decrease in the control group:” **This point has now been addressed accordingly in Figures 2 and 3.**

Also, was performance similar between groups post-warm-up?: **This point has now been addressed accordingly in Figures 2 and 3 and in the manuscript, for every variable.**

**Post hoc analyses revealed that, as hypothesized, performance in the EXP group was lower than in the CTRL group after the warm-up phase.**

**We have now added and made these effects more visible in the figures (using the symbol # to indicate significant post hoc differences in the post-test phase), as well as in the Results section.**

In the text, values during pre-tests are useless and redundant with figure 2: **We agree that the numerical values reported in the text and the graphical information provided in Figures 2 and 3 convey the same results. However, the text provides decimal precision that may not be directly visible in the figures, which we consider important for cross-study comparisons and potential future reproducibility analyses. At the same time, the graphical representation of these data facilitates a rapid and intuitive understanding of the findings. For these reasons, we believe it is valuable to retain both formats (and we would like to do it), as they serve complementary purposes in the presentation of our results. However, should the reviewer consider that this redundancy compromises the fluency and clarity of the manuscript, we would be willing to remove the values from the text if deemed necessary in a subsequent revision.**

L290. Use '.' and not ',' for values: **We agree with this remark, and this point has now been addressed accordingly in the manuscript.**

Table 2. One can question about the variability obtained for LF and HF. How can authors explain this huge inter-individual and inter-group variation? How can authors explain the lack of difference in pre-values between groups. These results are questionable.

**We thank the reviewer for raising this important point regarding the high inter-individual and inter-group variability observed in the LF and HF components. Several complementary explanations can be added:**

**First, differential responsiveness to psychophysiological techniques: The large pre-to-post variation across individuals likely reflects genuine differences in sensitivity to mental imagery and controlled breathing techniques. Some participants are inherently more responsive or “receptive” to such interventions, which leads to greater autonomic modulation, while others exhibit minimal or variable shifts. In our prior work, we too have observed substantial inter-individual variance in autonomic responses to similar protocols (Biéchy et al. 2021).**

**Second, dealing with the group divergence driven by adaptation to the intervention: the intergroup divergence may very probably result from the intervention itself: the CTRL group exhibited a drop in sympathetic tone (or an increase in parasympathetic dominance) following the standard warm-up, whereas the EXP group, by virtue of the psychophysiological additions, showed maintained or even enhanced sympathetic activation and reciprocal changes in parasympathetic indices. These processes of physiological adaptation would naturally widen group differences in LF/HF responses.**

**Third, intrinsic biological variability in HRV
It is also well recognized in the HRV literature that large inter-individual variability is the norm, particularly for spectral (LF/HF) metrics, due to differing baseline autonomic regulation, genetic factors, lifestyle, cardiorespiratory coupling, and measurement noise. For example, heritable factors have been shown to account for 47–64 % of HRV variance across individuals (Thayer et al. 2012; Golosheykin et al 2017; Schaffer et al. 2017). Moreover, methodological issues such as respiration rate, posture, and measurement imprecision further amplify variability in LF and HF estimates (Schaffer et al. 2017).**

**Given these points, we believe the observed variability is plausible and meaningful rather than artifactual. Nevertheless, and in adequation with other remarks, we will add a more explicit discussion of this issue in our limitations section, acknowledging that with a larger sample size or repeated measures one might better partition “responder” vs “non-responder” phenotypes. This also relates to the broader question and future perspective of systematically assessing imagery ability prior to implementing psychophysiological optimization protocols in tactical athletes, which we consider an important avenue for future research.**

**References:**

- **Simon Golosheykin, Julia D. Grant, Olga V. Novak, Andrew C. Heath, Andrey P. Anokhin, (2017) Genetic influences on heart rate variability, International Journal of Psychophysiology, Volume 115, 2017, Pages 65-73, ISSN 0167-8760.**
- **Julian F. Thayer, Fredrik Åhs, Mats Fredrikson, John J. Sollers, Tor D. Wager, (2012) A meta-analysis of heart rate variability and neuroimaging studies: Implications for heart rate variability as a marker of stress and health,Neuroscience & Biobehavioral Reviews, Volume 36, Issue 2, 2012, Pages 747-756, ISSN 0149-7634.**
- **Shaffer F, Ginsberg JP. An Overview of Heart Rate Variability Metrics and Norms. Front Public Health. 2017 Sep 28;5:258. doi: 10.3389/fpubh.2017.00258**

**Discussion.**

L387-397. The aim of the study is different than the aim presented in introduction. Similarly authors indicate comparing percentage change. It was not done. In addition, some new terms are used (dynamization). Please be consistent throughout the manuscript.

**We agree with this remark, and we have modified accordingly as follows (highlighted in yellow) to be consistent throughout the manuscript:**

**“****The primary aim of this study was to investigate whether incorporating mental imagery and conscious breathing exercises into a conventional warm-up routine could optimize cardiac autonomic modulation, muscular strength, and endurance performance in firefighters. To address this question, two groups performed the same physical warm-up, with the experimental group also engaging in psychophysiological techniques during the exercises. Cardiac autonomic activity was assessed through HR and HRV indices, while maximal voluntary isometric handgrip contraction, maximal push-ups, and abdominal plank duration were used to evaluate muscle strength and endurance. Our findings indicate that integrating mental imagery and controlled breathing elicited greater sympathetic activation and higher HR responses compared with the control warm-up alone. This modulation of the ANS was accompanied by attenuated declines in repeated maximal performance, suggesting that psychophysiological practices helped mitigate both central and peripheral fatigue. Taken together, these results support the integration of targeted psychophysiological strategies into operational warm-up routines to enhance readiness and performance in tactical populations such as firefighters.”**

L399-415. This part is unclear and is too briefly discussed. Authors indicate MVIC decrease is due to fatigue. How it it possible? A warm-up should limit fatigue. What does authors mean with repeating maximal and submaximal contractions. This repetition induce fatigue? So it means that authors have not measured any warm-up effects. Tests per se bias the results?

**We thank the reviewer for this constructive comment. We agree that the purpose of a warm-up is to facilitate performance and potentially reduce the onset of fatigue effects. In our study, however, the decrease in MVIC was not interpreted as an immediate effect of the warm-up itself, but rather as a consequence of the testing protocol, which required participants to repeat successive maximal and submaximal efforts (handgrip is a maximal effort, push-ups test is a repetition of submaximal efforts until exhaustion, plank is a sustained submaximal isometric contraction to exhaustion). Such repetitions are well known to induce both central and peripheral fatigue, independently of the warm-up condition (Tornero-Aguilera et al., 2022; McKenna & Hargreaves 2008).**

**The rationale of our design was precisely to evaluate whether supplementing a standard warm-up with psychophysiological techniques (mental imagery and controlled breathing) could attenuate this performance decline during repeated maximal tasks—thus paralleling the real operational context of firefighters performing multiple interventions during a duty shift. In this framework, the warm-up does not eliminate fatigue but may influence the rate or magnitude with which it develops. Our results showed that the experimental group maintained higher performance levels compared to controls, suggesting that the psychophysiological additions optimized physical performance, most likely by enhancing autonomic nervous system activity and corticospinal excitability, thereby counteracting the effects of fatigue rather than preventing it entirely.**

**Thus, the observed decrease in MVIC reflects the natural physiological cost of repeated maximal exertions rather than a failure of the warm-up per se, and the between-group differences clearly demonstrate the added value of the experimental condition. We will clarify this point in the revised manuscript to avoid ambiguity as following:**

***“******The observed decrease in MVIC should not be attributed to the warm-up itself but rather to the physiological cost of the testing protocol, which involved successive maximal and submaximal efforts (i.e. handgrip MVIC represents a maximal effort, push-ups entails submaximal efforts until exhaustion, and the abdominal plank-test involves a sustained submaximal isometric contraction to exhaustion) known to induce both central and peripheral fatigue (McKenna & Hargreaves 2008; Tornero-Aguilera et al., 2022). In this context, the warm-up—particularly when supplemented with mental imagery and controlled breathing—does not prevent fatigue but modulates its magnitude and physiological impact. Accordingly, the EXP group maintained higher performance levels than the CTRL group, suggesting that psychophysiological practices helped mitigate fatigue-effects, likely through enhanced autonomic regulation and corticospinal excitability.”***

L428. I still can't understand the hypothesis about performance decrements. A warm-up is not conducted to produce fatigue.

**We agree, the warm-up itself is not intended to produce fatigue (on the contrary, a warm-up is precisely intended to maintain a subtle yet antagonistic balance between increasing intramuscular and core temperature and limiting energy expenditure and fatigue!). Rather, our hypothesis focused on performance decrements induced by the repeated physical maximal tests, and whether psychophysiological techniques could attenuate this decline compared to the standard warm-up alone.**

L438-441. Same comment than for part 4.1.

**We thank the reviewer for this follow-up. As clarified above, the observed performance decrements do not reflect a direct effect of the warm-up, but rather the physiological cost of the repeated maximal tasks. Our rationale was precisely to test whether psychophysiological techniques could attenuate this decline compared to a standard warm-up, and we have revised the manuscript accordingly to make this point explicit.**

L446. Where do these 8minutes come from? Is it just from mental imagery or the combination of breathing and imagery?

**The 8 minutes refer to the cumulative time effectively dedicated to mental imagery within the EXP warm-up protocol. This time was distributed across the 13-minute warm-up sequence and was always performed with a physical exercise, and in some cases combined simultaneously with both controlled breathing and the physical task. Thus, the 8 minutes represent the total imagery practice time within the integrated protocol rather than imagery alone in isolation.**

**For the sake of clarity, we have modified accordingly as follows (highlighted in yellow) in the manuscript:**

***“Using a novel and pragmatic approach, the present study provides evidence that a brief intervention consisting of only eight minutes of mental imagery, cumulatively distributed across the 13-minute protocol and systematically performed alongside physical exercises, sometimes in combination with controlled breathing, is sufficient to enhance muscular performance during repeated maximal strength tasks in firefighters.”***

Part 4.3. Can authors make the link between sympathetic activation and performance (cardiac, muscle or endurance). The likely effects and applications are not so clear.

**Sympathetic activation increases cardiac output (via raised HR and contractility), redistributes blood flow to working muscles, and enhances neuromuscular excitation, thus supporting higher force output and delaying (or decreasing) fatigue-effects. In other words, by raising perfusion pressure and enhancing motor unit recruitment, a modest sympathetic upregulation can improve muscular performance under demanding tasks. Several recent studies report associations between sympathetic markers and performance metrics. For instance, the suppression of HRV (manifesting as reduced HF or elevated LF/HF ratio) is commonly observed when sympathetic dominance is engaged during intense exercise, reflecting increased sympathetic drive (Yugar et al. 2023, Addleman et al. 2024, Lassing et al. 2025). Also, resistance‐exercise protocols have been shown to produce sympathetic activation correlating with greater power output or strength maintenance (Addleman et al. 2024). In our study, the EXP group exhibited higher HR and shifts in HRV compatible with sympathetic upregulation when compared to CTRL. This aligns with their ability to resist the decline in maximal performance across repeated tasks. Thus, the data could support the hypothesis that psychophysiological techniques facilitated a more favorable autonomic state (sympathetic priming) that translated into better sustained performance.**

**References:**

- **Addleman, J. S., Lackey, N. S., DeBlauw, J. A., & Hajduczok, A. G. (2024). Heart Rate Variability Applications in Strength and Conditioning: A Narrative Review. *Journal of Functional Morphology and Kinesiology*, *9*(2), 93.** https://doi.org/10.3390/jfmk9020093
- **Lässing, J., Wegener, F., Höpker, N. *et al.* Heart rate variability response of intensity-matched strength training dependent on body position in females: a pilot randomized crossover study. *Sci Rep* 15, 32550 (2025). https://doi.org/10.1038/s41598-025-19817-7**
- **Yugar LBT, Yugar-Toledo JC, Dinamarco N, Sedenho-Prado LG, Moreno BVD, Rubio TA, Fattori A, Rodrigues B, Vilela-Martin JF, Moreno H. The Role of Heart Rate Variability (HRV) in Different Hypertensive Syndromes. Diagnostics (Basel). 2023 Feb 19;13(4):785. doi: 10.3390/diagnostics13040785.**

**For the sake of clarity, the following sentences have been added to the relevant paragraph in accordance with the reviewer’s remark.**

***“Increased sympathetic activation, reflected by elevated HR and shifts in HRV indices, likely contributes to performance maintenance by improving cardiac output and muscle perfusion, and increasing neuromuscular excitability. In the EXP group, this autonomic facilitation coincided with smaller declines in repeated maximal tasks, supporting a mechanistic link between sympathetic priming and sustained force and endurance performance.”***

L517-528. I disagree with the conclusion. imagery and breathing do not improves performance. It is not shown by statistics.

**We thank the reviewer for this remark. We agree that it would be an overstatement to speak of “improvement”; our results rather indicate an optimization of repeated performance, by mitigating the decline observed in the control group, rather than a net increase beyond baseline.**

**We have modified the manuscript accordingly (highlighted in yellow) to address this remark.**

***“Conclusion***

***This study is among the first to investigate the integration of psychophysiological techniques into firefighters’ warm-up routines. The experimental protocol involved repeated maximal and submaximal efforts performed to exhaustion, which inevitably resulted in performance decline across sequences. Our findings show that during the second repetition of these maximal tasks, the participants whose warm-up included mental imagery and controlled breathing exhibited a smaller decline in muscular performance than the control participants, while also exhibiting higher heart rate and HRV shifts indicative of increased sympathetic activation. This autonomic modulation is likely one of the mechanisms underlying performance optimization, through improved muscle perfusion and enhanced neuromuscular excitability. Given the repeated high-intensity demands characteristic of firefighting missions, these strategies may represent a cost-effective and time-efficient means of supporting autonomic readiness and sustaining operational performance in first responders.”***

Part 6. Some limitations could be added.

**We agree, and in line with this remark and considering the overall reviewing process, we have added the following Limitations and Perspectives paragraph to our manuscript.**

***“Limitations and Perspectives***

***This study has several limitations. Only 28 of the 34 participants were equipped with HRV sensors due to equipment availability, although all completed the physical tests. HRV indices—particularly LF and HF parameters—showed substantial inter-individual variability, likely reflecting differences in imagery ability, breathing control, and baseline autonomic regulation. Future studies should therefore examine individual characteristics more closely, especially imagery ability and responsiveness to imagery practice, which requires systematic evaluation of participants’ capacity to generate and perform motor imagery. Motor imagery could not be directly monitored during exercise execution in the absence of neurophysiological measures; laboratory-based approaches, such as fMRI, could help address this limitation. Furthermore, the 15-minute HRV test protocol necessitated a second warm-up to ensure participant safety before the subsequent muscular tests. This may have modulated the magnitude of observed effects, although the procedure was strictly identical across groups to prevent comparison bias. Finally, because the protocol involved repeated maximal and submaximal efforts to exhaustion, the observed performance decrements reflect the physiological cost of the testing sequence rather than a direct effect of the warm-up; thus, findings should be interpreted as relative group differences. Future research should aim to implement comprehensive HRV monitoring, evaluate long-term adaptations, and explore the operational transfer of such psychophysiological routines in first responder contexts.”***
